# Assessment of the Hardening Behavior and Tensile Properties of a Cold-Rolled Bainitic–Ferritic Steel

**DOI:** 10.3390/ma14216662

**Published:** 2021-11-04

**Authors:** Emilio Bassini, Antonio Sivo, Daniele Ugues

**Affiliations:** 1 Dipartimento di Scienza Applicata e Tecnologia, Politecnico di Torino, Corso Duca degli Abruzzi, 24, 10129 Torino, Italy; antonio.sivo@polito.it (A.S.); daniele.ugues@polito.it (D.U.); 2 Consorzio Interuniversitario Nazionale per la Scienza e Tecnologia dei Materiali (INSTM), Via G. Giusti 9, 50121 Firenze, Italy

**Keywords:** dual-phase steel, advanced high-strength steel, cold rolling, bainitic–ferritic steel

## Abstract

The automotive field is continuously researching safer, high-strength, ductile materials. Nowadays, dual-phase (DP) steels are gaining importance, since they meet all these requirements. Dual-phase steel made of ferrite and bainite is the object of a complete microstructural and mechanical characterization, which includes tensile and bending tests. This specific steel contains ferrite and bainite in equal parts; ferrite is the soft phase while bainite acts as a dispersed reinforcing system. This peculiar microstructure, together with fine dispersed carbides, an extremely low carbon content (0.09 wt%), and a minimal degree of strain hardening (less than 10%) allow this steel to compete with traditional medium-carbon single-phase steels. In this work, a full pearlitic C67 steel containing 0.67% carbon was used as a benchmark to build a comparative study between the DP and SP steels. Moreover, the Crussard–Jaoul (C-J) and Voce analysis were adopted to describe the hardening behavior of the two materials. Using the C-J analysis, it is possible to separately analyze the ferrite and bainite strain hardening and understand which alterations occur to DP steel after being cold rolled. On the other hand, the Voce equation was used to evaluate the dislocation density evolution as a function of the material state.

## 1. Introduction

From the early 1970s [1] until recent years, increasing safety and reducing car weight are two of the most critical design criteria adopted in car production facilities. New steels have been developed with enhanced formability, high strength, and good strain-hardening indexes to achieve such a challenging task. The final purpose of this intense research work is to build light vehicles that can withstand severe impacts, ensuring passenger safety. Several studies [1,2,3,4,5,6,7,8,9,10,11,12,13,14] indicate that the automotive field uses an increasing amount of advanced high-strength steels (AHS). Several alloys are enclosed in this class of steels, such as transformation-induced plasticity (TRIP) steels, twinning-induced plasticity (TWIP) steels, and dual-phase (DP) steels, the last ones being the most common. These steel grades show superior mechanical properties, which can be finely adjusted by varying the steel chemical composition or the heat treatment. Among AHS, dual-phase or multi-phase steels are gaining great popularity since they incorporate high strength, ductility, and toughness, which is a combination of properties that is not achievable with conventional single-phase steels [11].

The contemporary presence of two phases brings good formability and excellent mechanical properties, making DP steels a good choice in the automotive field. This material grade consists of a mixture of ferrite (soft phase) and a second reinforcing phase that can be martensite or bainite or even a combination of the two. These steels are typically obtained via cold rolling of metal sheets, which is followed by an intercritical annealing. Thanks to this thermomechanical treatment, the initial ferritic/perlitic microstructure is modified by the formation of austenite during the soaking at the intercritical temperature. Consequently, austenite is transformed into martensite or bainite due to the subsequent quenching or isothermal transformation [4,5,6,7,8,10,11,12,14,15,16,17,18,19,20,21]. Nowadays, the most diffused DP steels contain martensite or bainite in low volume fraction, i.e., between 5% and 15% [22].

Conversely, this paper assesses the mechanical properties and formability of DP steel containing a 50% volume fraction of Bainite. The main objective here is to clearly understand how cold rolling will affect the anisotropy levels of the DP steels. At the same time, this material will be compared with traditional SP steel. Nevertheless, the only way to make the two steels comparable was applying a strong cold rolling to the SP steel, i.e., a 50% thickness reduction. Then, the comparison was possible, but this also evidenced that the SP steel was already close to its formability limit.

Nevertheless, the drawability of DP steels has been demonstrated to be lower than traditional steels due to their low r-value (anisotropy), limiting their application [14,23]. Even if, according to the work of Ashrafi, drawability can be enhanced using specific heat treatments such as a double-step annealing process [23]. 

At the moment, to the author’s knowledge, no work describes the anisotropy and the hardening behavior of a DP steel with such a high-volume fraction of bainite. Despite this, to build a solid comparison between the two material categories, some information has been found regarding more traditional steels and will be presented below. 

According to Toribio [24], steel anisotropy is always altered when a certain degree of strain hardening is introduced, which is valid for single or dual-phase materials. For example, in pearlitic steels, the plastic deformation will create a textured material, aligning the lamellar islands along the rolling direction. Furthermore, because of the anisotropy level of cold working, cracks will propagate along the rolling direction when failure happens. Similarly, Magnabosco [22] evidenced that the crystallographic texture introduced by the metalworking has dramatic effects on the mechanical properties. These notably change if measured in the longitudinal or transversal orientations related to the rolling direction. In other words, the expected value of anisotropy changes as the amount of cold work is increased. Finally, according to Komissarova [25], sometimes, a certain degree of anisotropy could even be helpful. This fact is true when the anisotropy in mechanical properties promotes equalization of the different actions of different forces occurring under conditions of complex loading of parts.

Conversely, this condition can be adverse when the anisotropy reduces mechanical properties in individual directions. It is difficult to find an expected or “correct” anisotropy value from the literature for SP or DP steels for all these reasons. Consequently, these values should be calculated every time it is needed or when the effect of plastic deformation on overall mechanical properties is unknown, as for the DP steel discussed in this paper.

Due to the contemporary presence of two phases, DP steels show high initial strain hardening and a high tensile-yield strength ratio, which leads to a relatively high ductility [26]. In particular, they offer a higher work-hardening rate, especially at low strains [14,26]. The volume fraction of ferrite strongly influences ductility. Saedi [6], in his work on the ferritic–martensitic 4340 steel, demonstrated that ductility increases until the volume fraction of ferrite is below 34%; then, it starts decreasing. On the other hand, the same author [20] showed that the same steel might have enhanced tensile strength and ductility by adding a 25%_vol_ of bainite. Furthermore, the shape of the two phases has an essential role in altering mechanical properties, especially the toughness of the steel [27,28].

Many efforts have been made to describe the strain-hardening behavior of metals via constitutive equations. Hollomon and the Crussard–Jaoul formulations are mainly adopted for this task [23,29,30,31,32]. These models are known for modeling the true strain–true stress curves with two indices, i.e., n and K. Typically, a material with a high value of n is preferred if intense plastic deformation is needed. Furthermore, a high value of n indicates that the material has a higher strain-hardening rate; thus, it can withstand severe deformation before reaching instability. On the other hand, n can also be correlated to the elastic spring-back effect. Typically, in steels, an inverse relationship is observed between the two, but DP steels seem not to follow this general rule [15]. Although the previously cited methods are versatile, they demonstrated relatively ineffective describing DP-hardening behavior since an exponential function cannot fit their σ-ϵ curves. In particular, during the plastic deformation, many DP steels show two or even three different strain behaviors [31].

According to the literature, the strain-hardening rate of DP steels is higher than classical single-phase steels [33]. Ashby, who first studied this behavior, introduced the concept of Statistically Stored Dislocation (SSD) and Geometrically Necessary Dislocation (GND) to explain the higher strain-hardening rate observed [33]. More recently, this was explained, considering that plastic deformation is principally located in the soft ferrite, while harder phases maintain elastic condition up to necking. This condition is particularly evident in DP steels made of a mixture of ferrite and martensite and is also appreciable for those containing low volume fractions of bainite. Thus, this paper will also aim to confirm these hypotheses by manipulating the stress–strain curves generated with the tensile test for a DP steel grade characterized by a higher volume of bainite. In particular, it was demonstrated that DP steels with ferrite and a 35% volume fraction of bainite show the highest strain hardenability [19]. However, still, no papers investigated a DP steel with 50% of bainite.

The DP steel is characterized in two different states, as provided and after a 9% thickness reduction after cold rolling. The results are compared to a standard product as C67, which was used as a benchmark. The calculated hardening indexes within this paper could also be helpful for modeling this material’s nonlinear plastic behavior. At the same time, knowing the anisotropy levels and the bending behavior of this DP steel could be interesting for those interested in using a more efficient steel grade by tailoring their existing manufacturing process chains. 

## 2. Materials and Methods 

### 2.1. Materials and Samples Preparation

Table 1 shows the average chemical compositions of the DP steel and C67, respectively, expressed in wt% according to the manufacturer datasheets. The results were in good agreement with EDS analysis performed with an Oxford instrument probe. On the other hand, the carbon levels were double checked with Leco analysis (Leco Corporation, St. Joseph, MI, USA) using the combustion technique. Again, the results were in good agreement with data provided from the steelmaker. 

This DP steel is characterized by the contemporary presence, in equal parts, of ferrite and bainite. This peculiar microstructure is obtained with a controlled thermomechanical treatment performed during the hot rolling process. More specifically, the as-received material was obtained via a controlled hot rolling process. The initial part of the process must be performed above A_3_, i.e., 883 °C. In fact, the suggested temperature for this step is 1035 °C. The temperature within the mills is strictly controlled. After the initial thickness reduction, the steel is locally maintained between the bainite start (Bs) and bainite finish transformation temperatures (i.e., between 618 and 542 °C). The longer the soaking time within this temperature range, the higher the final bainite volume will be found. The correct time to get 50% bainite was considered 12 min.

In this paper, DP steel was characterized in the as-received condition (also indicated as DP 5.5 mm) and after a 9% thickness reduction via cold rolling. On the other hand, C67 is a fully pearlitic, low-alloyed steel obtained after traditional hot rolling and slowly cooled down to room temperature. Then, the obtained sheet metal was cold rolled to get a 50% thickness reduction. This procedure was necessary to make the two materials comparable. More precisely, the materials were work-hardened to achieve the minimum yield stress requested by the Euro NCAP standard for automotive B-pillars construction [34]. As a result, the 9% thickness reduction of the DP steel was enough to satisfy the standard. Furthermore, this value was obtained thanks to a complete work-hardening assessment with thickness reduction from 5 to 80%, which will be discussed in a future research paper. 

All samples were in sheet form. The as-received samples thickness was 5.5 mm. After cold rolling, both DP (9% deformation) and C67 (50% deformation) showed a 5 mm thickness. Therefore, the microstructure of these three samples was assessed in the longitudinal and transverse directions. According to the standard metallographic procedure, specimens have been cut, mounted in resin, and ground with SiC paper up to 2400 grits. Then, samples were polished with diamond pastes up to 1 µm. After each polishing step, samples were rinsed with abundant water and then cleaned with ethanol. Microstructures were revealed after etching with Nital 4%. The metallographic observation was performed with light optical microscope Leica MEF4, while the finest features and phases were displayed with scanning electronic microscope (SEM) type MEB Leo 1450 VP.

### 2.2. Tensile Tests

Tensile tests were performed according to ISO 6892 standard using a tensile machine Zwick Z100 equipped with a 100 kN loading cell and a macro extensometer, which measured elongation up to samples failure. Tensile tests were performed on specimens with three different metallurgical orientations, i.e., parallel, transversal, and 45° slant with respect to the rolling direction. Each condition was evaluated with 5 different samples. Elongation at maximum strength and elongation at break were measured during the tensile test. In addition, some broken specimens were used to calculate the normal (*R_m_*) and planar (Δ*R*) anisotropy for both steels according to ASTM E517-2010 standard using Equations (1) and (2), respectively.

(1) $$R_{m}=\frac{r_{0}+r_{90}+2r_{45}}{4}$$

(2) $$\Delta R=\frac{r_{0}+r_{90}-2r_{45}}{2}$$

where *r* is calculated following the referenced work [22] using the following formula:

(3) r=ln(ww0)ln(tt0).

Here, *w*_0_, *t*_0_, *w*, and *t* represent the width and the thickness of the tensile samples before and after the tensile test, respectively. The subscript of *r* indicates the metallographic orientation for which the ratio was calculated. Then, broken samples were observed under stereomicroscope Leica MZ6 and with SEM. 

### 2.3. Three-Point Bending Tests

Tests were conducted in load control mode using the same machine previously described; the cross-head displacement assessed the deformation. In addition, samples were prepared to observe possible differences among the three metallurgical orientations. Since no sample reached a complete break, the crack path was assessed with a stereomicroscope. 

**Figure 1:**
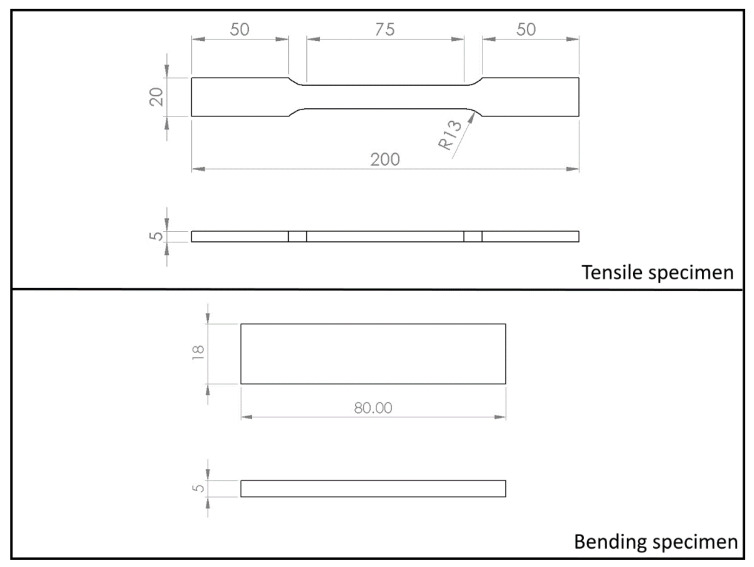
Schematics showing the actual size of tensile and bending specimens used within this research work.

Figure 1 shows the specimens schematic with the suitable size expressed in mm.

## 3. Results

### 3.1. Steels Microstructure

**Figure 2:**
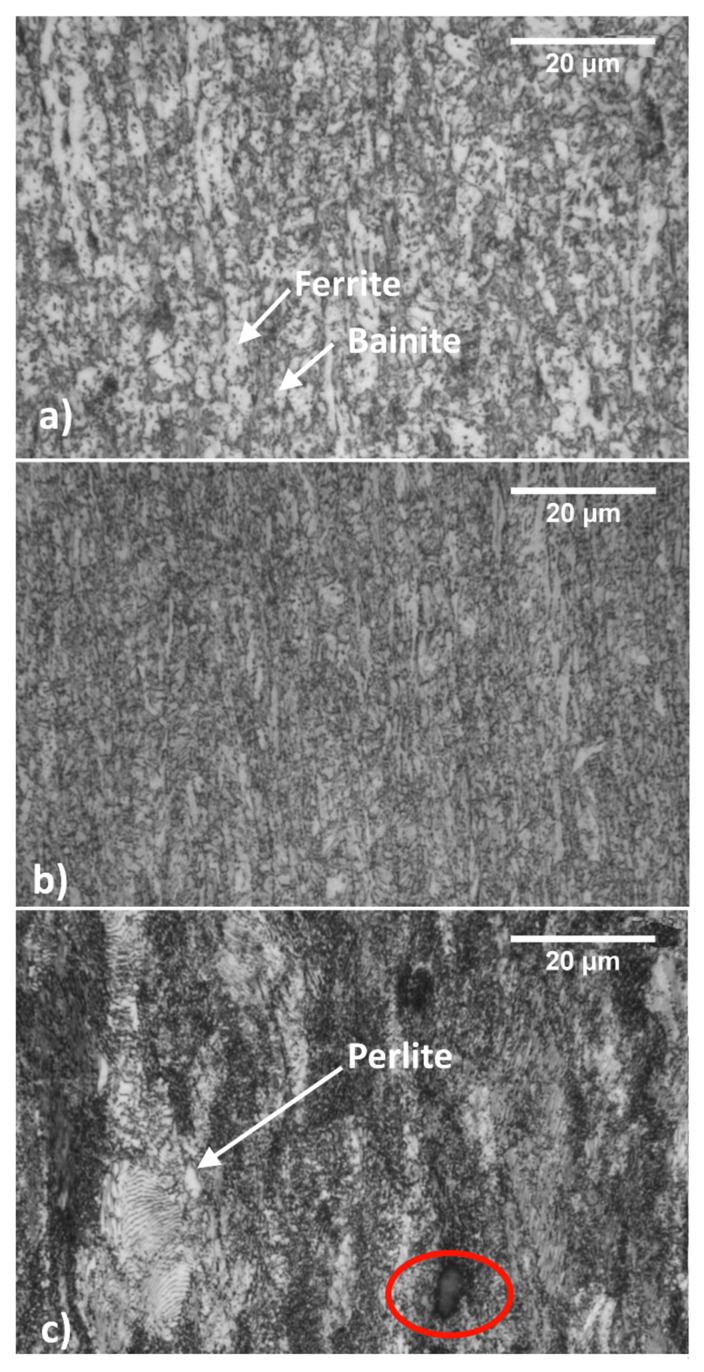
Steels microstructures observed with LOM: as received (hot-rolled) DP steel (**a**); 9% cold-rolled DP steel (**b**) and C67 (**c**) ferrite is less reactive toward the etching, thus remaining whiter.

Figure 2 shows the microstructures of the steels with longitudinal orientation observed with a light optical microscope (LOM). Figure 2a,b show the DP steel as received and after 9% cold working. The dual-phase structure is already visible with the LOM; ferrite appears brighter, while bainite is darker. After cold working, the microstructure appears finer.

The DP steel microstructure is homogenous. Longitudinal samples show typical plastic flow lines but only weak microstructural orientation. On the other hand, C67 contains only perlite and pro-eutectoid carbides, as evidenced in Figure 2c with red ellipses. Since C67 underwent a 50% work hardening, its microstructure shows an evident orientation along the rolling direction. A white arrow points to a still visible pearlitic grain.

**Figure 3:**
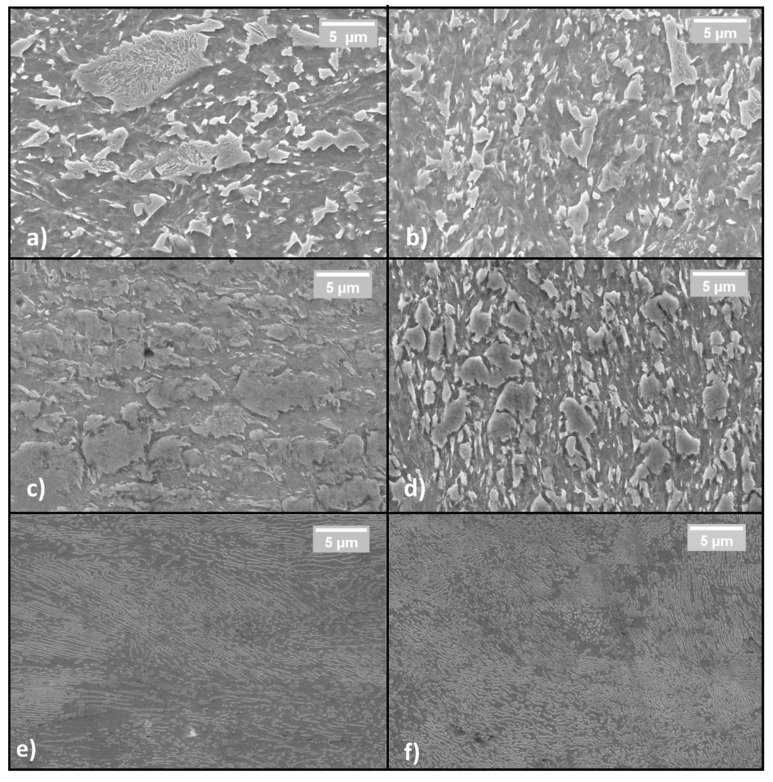
Sample microstructures in the longitudinal direction observed with SEM: (**a**,**c**,**d**). Sample microstructures in the transversal direction (**b**,**d**,**f**); from top to bottom: As-received DP steel (**a**,**b**); cold-rolled DP steel (**c**,**d**); C67 (**e**,**f**).

Figure 3 shows the SEM pictures, which better identify all the previously mentioned micro constituents. The left and right sides of the picture describe the samples in the longitudinal and the transversal orientations, respectively.

More specifically, Figure 3a,b show the DP steel in its as-received condition. Bainite is brighter and ferrite is darker. Figure 3c,d show the same material after cold working. The bainite islands appear almost unchanged in size but closer one to the other. This fact indicates that only ferrite is deformed at a low strain-hardening level while bainite is exclusively shifted inside the softer matrix. Finally, Figure 3e,f show the C67 pearlitic microstructure with a strong orientation along the rolling direction. 

### 3.2. Mechanical Characterization

**Figure 4:**
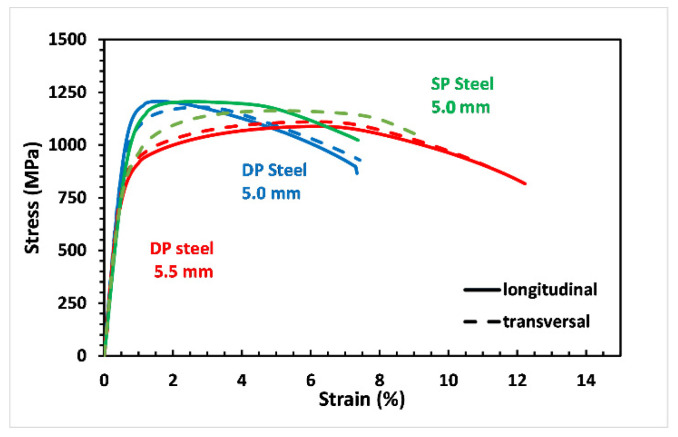
By a thickness reduction of 9% mechanical properties of DP steel change, higher yielding and maximum strength are accompanied by an elongation reduction (see the blue line).

In its as-received state, the DP steel was used as a reference to observe the increment in tensile properties reducing the thickness from 5.5 to 5.0 mm (see Figure 4). Then, both the results were compared to the C67 curves used as a benchmark. The picture shows that the mechanical properties of the DP steel in the as-received state are practically the same since the degree of anisotropy is too tiny to give significant divergences between the two curves. However, as the cold rolling is applied, a higher degree of anisotropy arises, leading to a stronger deviation between the curves; see the solid and dashed blue lines.

As-received DP steel showed very similar mechanical properties independently of the sample’s metallurgical orientation. With a 9% reduction in thickness, cold-rolled DP steel offers a markedly increased tensile resistance, especially in the longitudinal direction. A decrease in elongation at break is also observable. More specifically, for the longitudinal samples, the yield stress and the stress rupture increased from 685 to 982 MPa and from 1100 to 1212 MPa, respectively. At the same time, elongation decreases from 12.2 to 7.5%. Similar results were also observed in the other metallurgical orientation. Furthermore, it is possible to observe that DP steel and SP steel share almost the same mechanical properties after cold working. More precisely, both the materials have better mechanical properties along the longitudinal direction, but differences are more evident in the C67 specimens.

Furthermore, normal (*R_m_*) and planar (Δ*R*) anisotropy for C67 and cold-rolled DP steel were calculated and summarized in Table 2. Finally, the calculation was performed accordingly with the procedure described above, in Section 2.2 using Equations (1) and (2). 

The mean normal anisotropy, *R_m_*, is strongly related to the sheet-forming capability of a material. The higher *R_m_*, the better the formability of a material. In this case, both steels have similar and relatively high *R_m_* values, indicating good formability. Δ*R*, on the other hand, has great importance when deep drawing is involved. A material with Δ*R* equal to zero will not show “earing” at the end of the forming process; by contrast, values different from 0 indicate that earing will occur, as its position (related to the rolling direction) is a function of the Δ*R* sign. According to this, DP steel and C67 will show a similar tendency to form earing after deep drawing in the former case at 45°, whereas for the latter, it is at 0 and 90° to the rolling direction. From this analysis, it can be concluded that both steels can be used for sheet forming, while their usage for deep drawing should be discouraged.

**Figure 5:**
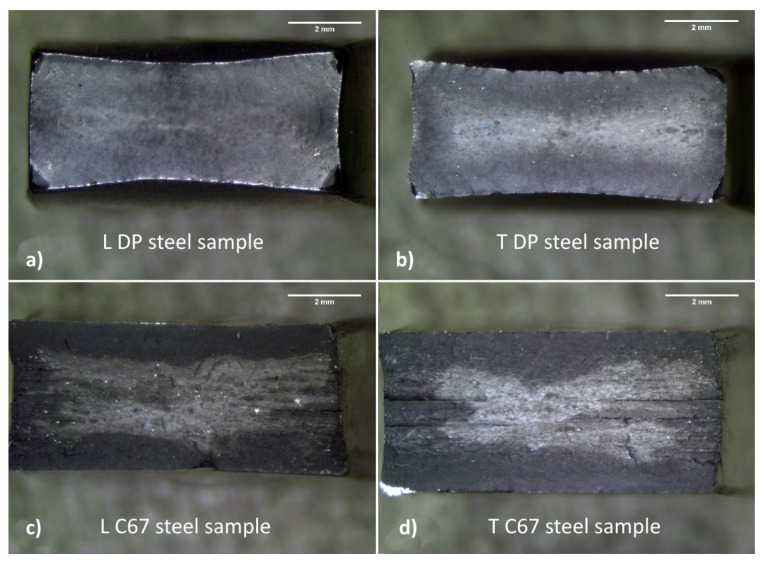
Broken tensile samples under stereomicroscope; DP steel (**a**,**b**) shows more deformed edges compared to C67 (**c**,**d**).

The differences in calculated anisotropy values are also reflected in the appearance of fractured samples, as visible in Figure 5. Longitudinal (L) and transverse (T) specimens are shown in the left and right parts of the picture, respectively. DP steel samples (Figure 5a,b) underwent an intense deformation at the edges with a progressive loss of the initial rectangular shape regardless of the metallurgical orientation. C67 samples, on the other hand, show only a slight distortion of the edges. C67 (Figure 5c,d) shows delamination among the fibers produced by the plastic flow. Such decohesion appears more clearly if samples are observed with SEM. Furthermore, it was noticed that no apparent differences appear in the fracture by changing the orientation of the samples. For this reason, only longitudinal samples will be further observed at the SEM.

**Figure 6:**
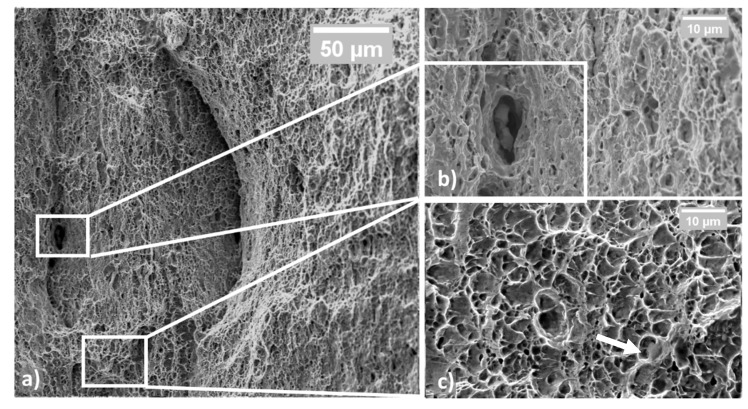
Fractured surface in cold-rolled BF steel samples; the appearance at low magnification is mainly ductile (**a**); some secondary phase was found, i.e., carbides (**b**); at high magnification, dimples are visible (**c**).

Figure 6a shows DP steel failure surfaces, which are characterized by ductile behavior. The pictures were taken away from the shear lips to better appreciate the presence of dimples, micro-voids, and any cleavage planes if present. The fracture was fully ductile, and it was caused by the coalescence of micro-voids formed upon the necking of the tensile sample. The sample was also observed throughout the entire surface to identify any sign of brittle fractures, such as cleavage plains, which were not detected. Some coarser particles were also observed, as indicated in Figure 6b. Despite this, DP steel did not show any delamination point where the plastic flow took place. Eventually, Figure 6c shows the dimples produced by the detachment of the dispersed secondary phases from the softer matrix. In the specific case of this DP steel, the particles responsible for the dimple formation can be both V or Mo primary carbides and the ϵ carbides contained in the bainitic islands. In the third panel, a white arrow underlines a trapped ϵ carbide located at the bottom of a dimple. 

**Figure 7:**
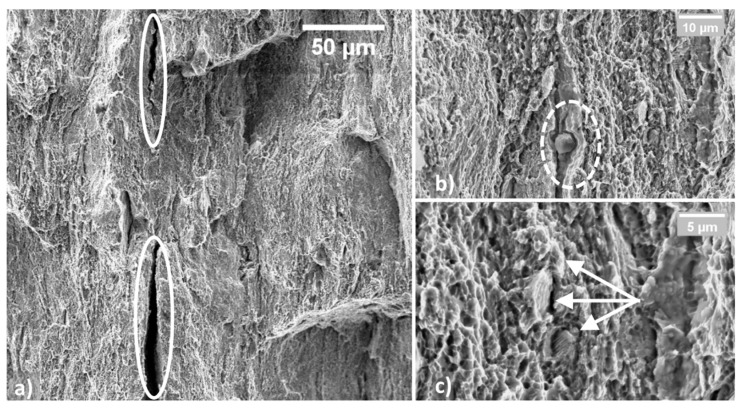
Fractured surface of tensile C67 sample; large areas appear as delaminated (**a**); secondary phases, such as pro-euctectoidic carbides were found (**b**); far away from delaminated areas, the fracture is ductile, and perlite grains are still visible (**c**).

On the other hand, Figure 7 shows the fractured surface of a C67 sample. In Figure 7a, white ellipses indicate where decohesion among the planes occurred. This fracture mode could also be aided by pro-eutectoid carbides, as evidenced in Figure 7b. These particles were responsible for raising the stress level locally during the tensile test. In this condition, the perceived stress level was higher in some isolated regions than the material ultimate tensile stress.

Consequently, deep grooves formed throughout the entire fractured surface. In these regions, little or no plastic deformation occurred, giving the impression of a more brittle fracture. Nevertheless, far away from the delaminated planes, fracture appears predominantly ductile with fine dimples. Some perlite grains are still observable at very high magnification (20,000×), as shown in Figure 7c. 

### 3.3. Bending Tests

**Figure 8:**
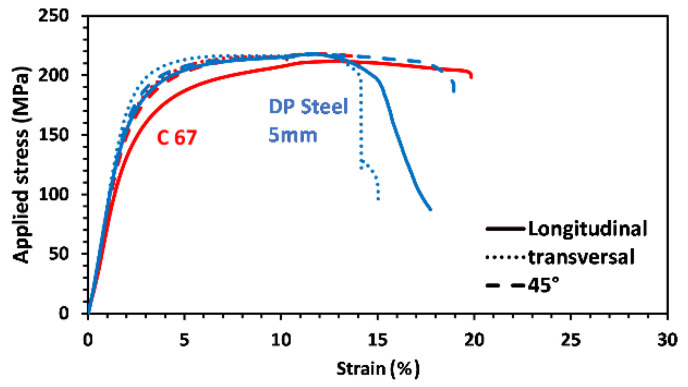
Bending curves of DP steel and C67 samples; each curve represents a different metallurgical orientation.

Bending tests are beneficial for simulating the material behavior during the manufacturing process requiring high plastic deformation. Figure 8 shows the curves obtained for the two materials. As can be seen, both materials can withstand similar stresses, despite the DP steel appearing less deformable, the 45° orientation being the only exception. More details are given in the next paragraph.

**Figure 9:**
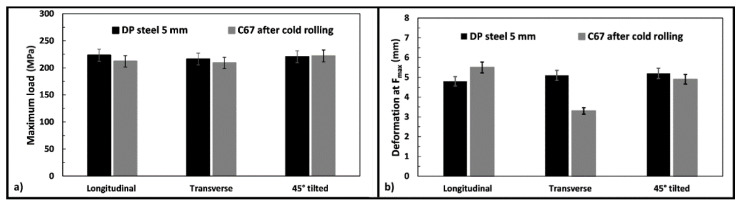
Averaged data from all the bending tests: maximum load measured (**a**) and maximum deformation observed (**b**), respectively.

Figure 9a shows the maximum load measured for the two steels, and the DP one has slightly higher values in the longitudinal and transverse direction. At the same time, only negligible results were observed in the third metallurgical orientation.

**Figure 10:**
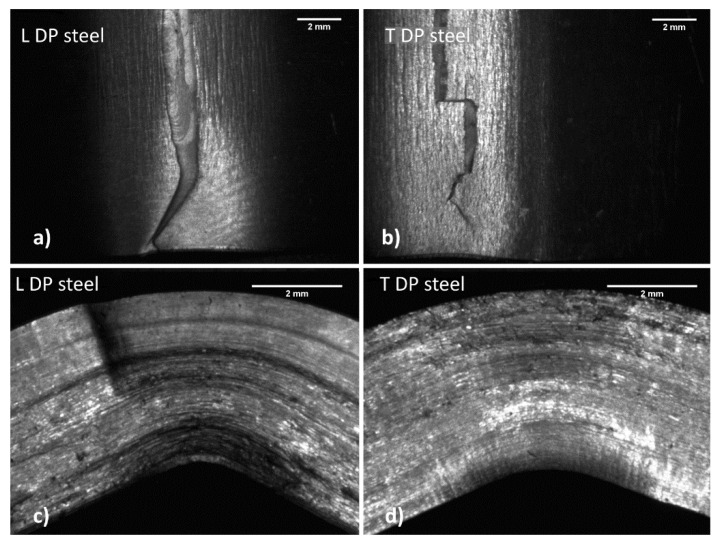
Both longitudinal (**a**,**c**) and transversal (**b**,**d**) DP steel samples show a small crack that travels into the resisting section with difficulty.

**Figure 11:**
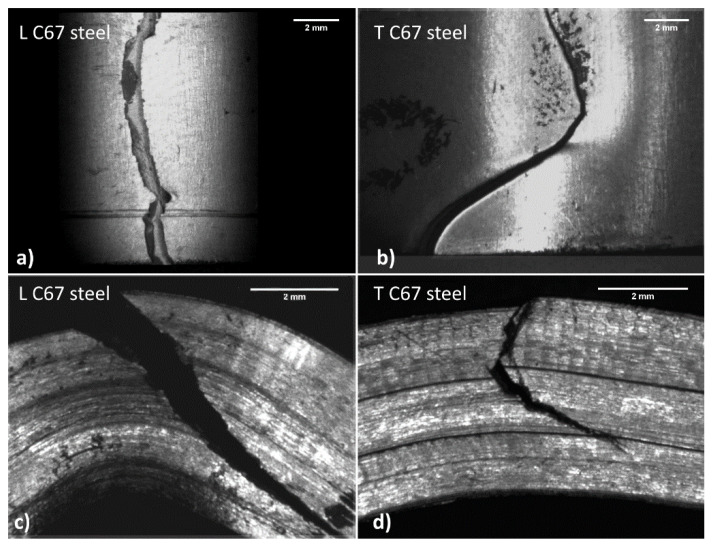
Both longitudinal (**a**,**c**) and transversal (**b**,**d**) C67 samples show a large crack across almost the entire resisting section.

Figure 9b compares the obtained deformation at maximum strength for the two products. In this case, the sample orientation has a substantial impact on the results. For example, C67 shows higher deformations in the longitudinal direction, while lower values correspond to the other rolling orientations. After the bending test, samples were observed with a stereomicroscope, since they were deformed but not wholly fractured. Figure 10 and Figure 11 show some of the most relevant cases observed. Longitudinal and transversal samples are described in the left and right parts of the pictures, respectively.

Samples observation (Figure 10) suggests that the two materials behave differently during bending. In DP steel samples (top view), the main crack nucleates at the surface in traction and presents several wrinkles due to plastic deformation. The crack tends to move only for a short path, which is small compared to the entire resistant section.

On the other hand, C67 (Figure 11) shows the main crack that quickly propagates through all the resistant sections after nucleation at the surface in traction. Crack moves through a stair-step-like delamination process. The crack initially moves along the interfaces among the material fibers in traction. These interfaces, as previously said, are due to the plastic flow undergone during hardening. Once the resistant section is drastically reduced, the crack can also travel in the material fibers, resulting in compression. Wrinkles due to plastic deformation are much less evident, since all deformation energy is dissipated during crack propagation. 

## 4. Discussion

### Crussard–Jaoul (C–J) and Voce Analysis of the Two Steels

**Figure 12:**
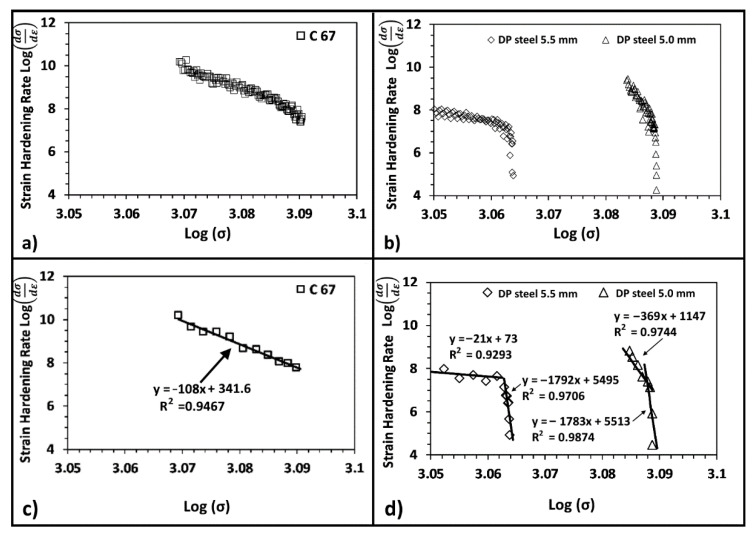
Strain-hardening curve for C67 (**a**) and for DP steel (**b**); all experimental data are shown. Same plots but with fewer experimental points for better representation (**c**,**d**).

DP steel’s behavior can hardly be modeled with traditional equations since plastic deformation occurs in two stages [23,31]. For this reason, DP steels are typically evaluated with other models, of which Crussard–Jaoul (C-J) is the most common [1,31,35]. The principal limitation of the Hollomon equation is the assumption that steel has a constant hardening exponent (n) along with the entire tensile test. The C-J analysis introduces the strain-hardening rate, i.e., $\frac{d\sigma }{d\varepsilon }$, which is plotted in logarithmic form against the true stress or the true strain. This data elaboration allows accounting for variation in the hardening behavior. Using the C-J analysis and plotting the logarithm of the strain-hardening rate against the logarithmic true stress, it is possible to separate the plastic deformation of the softer ferrite from that of the bainite. A knee in the curves describing the deformation process indicates the onset of plastic deformation of the harder phase. Figure 12a,b show the strain-hardening rate curve for an SP and an DP steel, respectively. In the first case, the whole material deforms homogeneously; hence, a linear single stage is visible, and the interpolating curve has been plotted in Figure 12c. On the other hand, DP steels have a discontinuity point due to the onset of bainite deformation. For this reason, two distinct interpolations have been performed for each curve part.

**Figure 13:**
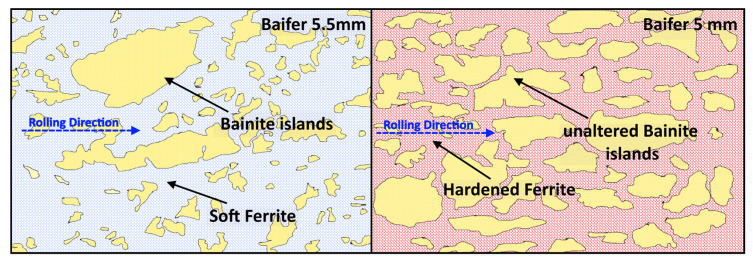
Unaltered bainite islands were shifted inside the hardened matrix. The whole strain hardening is withstood by ferrite (light blue when unaltered, red when hardened).

According to the literature, [1,33,35,36], the strain-hardening rate of dual-phase steels at low stress (very near to the yielding) is higher with respect to the single-phase one. In this work, the SP steel has a higher strain-hardening rate than the DP one. This fact could be related to the substantial thickness reduction adopted for C67 samples. This procedure, as mentioned above, was necessary to match the mechanical properties of the DP steel. On the other hand, the SP steel shows a single decreasing trend from yielding up to maximum load. Once bainite starts to deform plastically, the strain-hardening rate drops, and the curve shows an abrupt increase in slope. The thickness reduction of the DP steel also influenced the strain-hardening rate behavior. As shown in Figure 12b, its hardening rate increases by 20% after strain hardening the material. The increase in the σRσ0.2 ratio, progressively closer to 1, is also reflected in the strain-hardening rate plot: indeed, the chart shows a higher concentration of experimental points in a narrow range of loads. The slope of the curve related to the work-hardened DP steel is much higher than the others. The critical load beyond which the change in slope is located also shifted toward higher applied loads. It is noteworthy that the slope of the second part of the two curves is similar, as confirmed by the interpolation of experimental data. This fact indicates that bainite only went through a limited alteration due to the cold rolling process. At the same time, the most significant part of the work hardening is withstood by the softer phase, as indicated in the following schematic of Figure 13.

According to the literature and micrographies, it can be concluded that bainite islands are only shifted closer to one another but without being altered in shape, dimension, or mechanical properties [5,7]. On the other hand, softer ferrite is forced to shear under the cold rolling load, which leads to its strain hardening following the work of Tian [37]. Furthermore, the higher proximity of the bainite islands is very likely to hinder the slip of dislocations, which causes an overall increase in strength and a loss in ductility. This phenomenon is also predicted by the SSD and GND theory [1,38,39,40].

The previous hypothesis can be further supported using a modified version of the Voce Equation (4), as suggested by Xie in [29]. Indeed, coefficients of the equation can be directly related to several microstructural features of the material. Thus, the starting point is interpolating the true stress–true strain curves via nonlinear fitting using Equation (4).

(4) $$S=q\left[1-exp\left(-K_{2}Me^{p}\right)\right]/K_{2}$$

*S* is the true stress, *M* is Taylor’s factor and equal to 3, and *e* is the true strain. On the other hand, *q*, *K*_2_, and *p* are the calculated fitting coefficients. According to the literature [18,26,29,41,42,43], these parameters can be directly correlated to the microstructural features of the material, such as the density of dislocations and their resulting mean free path. More specifically, *q* is inversely proportional to the dislocations free path *λ*, as indicated in Equation (5).

(5) $$q\sim \frac{1}{b\lambda }$$

where *b* is a constant equal to 2.1.

*K*_2_ is related to the decrease in dislocations due to their annihilation or absorption by the sub-boundary [29,43]. The higher the starting density of dislocations, the higher the annihilation effect during the tensile test; hence, the higher *K*_2_. Finally, *p* can be assimilated to the Hollomon strain-hardening exponent *n*. Namely, *p* and *n* tend to coincide if *K*_2_ assumes minimal values. In this case, the modified Voce equation became practically indistinguishable from the Hollomon one.

**Figure 14:**
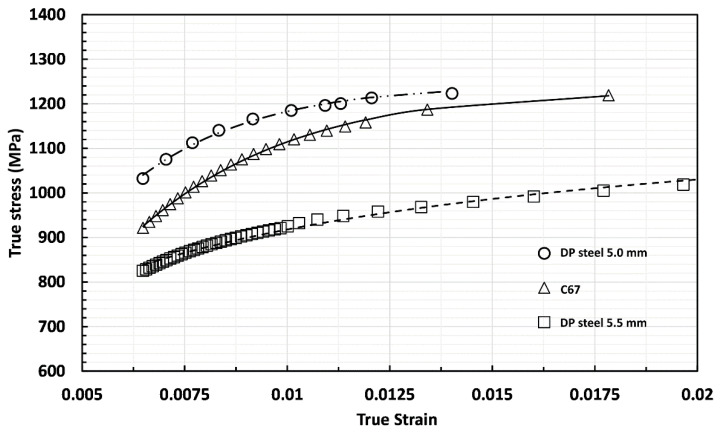
True stress–true strain curves for C67 and DP steel. Scattered symbols are experimental values, while lines are the calculated curves using the Voce equation.

Table 3 shows the calculated *p*, *K*_2_, and *q* coefficients with the obtained *R*^2^ values for the resulting interpolations shown in Figure 14 and the computed dislocations mean free path.

The mean free path results reported in Table 3 are in good agreement with those found by Bergstrom for a DP steel containing martensite after cold working, i.e., values ranging between 0.03 and 0.3 µm depending on the assessed work-hardening level [33]. 

Following the hypothesis proposed in the previous paragraph, C67 and cold-rolled DP steel have a very high value of *q* and *K*_2_, indicating a high density of dislocations provoked by the cold-rolling process itself. Even though DP steel was only slightly deformed with respect to C67, its density of dislocations and the mean free path are higher and shorter, respectively. This fact can be explained keeping in mind that the DP steel contains bainitic islands, which can hinder the slipping dislocation (leading to a lower value of *λ*). This reduction of the free mean path is particularly evident for the cold-rolled DP steel where the contemporary presence of a denser population of bainitic islands and the dislocation interactions push *λ* down an order of magnitude. The GND presence in this steel may explain the intense dislocation density change passing from the as-received to the cold-rolled state. 

## 5. Conclusions

A DP steel has been characterized in its as-received condition and after a 9% cold-rolling process. The exact characterization was also performed on cold-rolled steel (C67), undergoing a 50% thickness reduction to achieve similar mechanical properties. C67 was used as the reference material. The characterization performed assessed both the material’s microstructure and mechanical properties. In particular, tensile and bending strength were evaluated, and great attention was also given to the hardening behavior of the two steels.

At the end of the characterization, the following main conclusions can be drawn:

To achieve comparable mechanical properties with the DP steel, C67 was work hardened up to 50%, corresponding to the material-forming limit. All tests performed within this work confirmed this hypothesis. In particular, fractography shows strong decohesion among the material fibers caused by the plastic flow. The DP steel, on the other hand, received only a slight amount of deformation. 

The bending test is better tolerated by the DP steel samples even though cracks formed in most solicited regions of the samples. The complex crack path in the SP steel was caused by the high degree of anisotropy coupled with the orientation of pearlitic islands.

Since C67 is an SP steel, it can be described with traditional models, e.g., the Hollomon equation, while the DP steels need a more complex treatment of the true stress–true strain curve, and the C-J model is an excellent fitting model. Furthermore, the C-J analysis gives essential information about the phenomenon that took place during the strain hardening. In particular, it is possible to separate the plastic deformation of ferrite from bainite. Furthermore, bainite is only slightly altered by the cold-rolling process, as ferrite is the most altered phase.

According to the modified Voce equation, DP steel contains a higher density of dislocations due to the presence of GND with respect to C 67 steel. Moreover, the rate with which dislocation density increases due to the cold-rolling process is very high, as can be observed from comparing as-received DP steel with the 9% cold-rolled one.

## Figures and Tables

**Table 1:** Average chemical composition of DP steel and C67 expressed in wt% according to manufacturer datasheets.

	C	Si	Mn	Al	Cr	V	Mo	Fe
DP Steel	0.09	0.15	1.45	0.02	1.25	0.12	0.4	Bal.
C67	0.65	0.15	0.6	-	-	-	-	Bal.

**Table 2:** Normal (*R_m_*) and planar (Δ*R*) anisotropy, as calculated on broken tensile samples.

Material/Condition	R0	R90	R45	*R_m_*	Δ*R*
C67 (cold-rolled)	0.66	1.15	0.75	0.83	0.16
DP steel (as-received)	0.63	0.64	0.81	0.71	−0.15
DP steel (cold-rolled)	0.69	0.69	0.87	0.78	−0.18

**Table 3:** Fitting parameters of Equation (4), calculated dislocations mean free path, and accuracy of the interpolation expressed via *R*^2^.

Material	*q*	*K*_2_	*p*	*λ* (m)	*R* ^2^
C 67	2.79 E^6^	227.2	1.228	0.17 10^−6^	0.9998
DP steel 5.5 mm	4.39 E^7^	3.682	0.4388	0.10 10^−7^	0.9954
DP steel 5.0 mm	3.01 E^6^	242.2	1.189	0.15 10^−6^	0.9946

## Data Availability

All the data and results supporting this research paper are already presented within this publication.

## References

[1] Jiang Z.H., Guan Z.Z., Lian J.S. (1995). Effects of Microstructural Variables on the Deformation-Behavior of Dual-Phase Steel. Mater. Sci. Eng. A—Struct. Mater. Prop. Microstruct. Process..

[2] Saeidi N., Karimi M., Toroghinejad M.R. (2017). Development of a new dual phase steel with laminated microstructural morphology. Mater. Chem. Phys..

[3] Zhang Z., Cao J., Zhong Z., Zhou X., Chen W., Yang Y. (2017). Tensile deformation behavior of high strength anti-seismic steel with multi-phase microstructure. J. Iron Steel Res. Int..

[4] Tang C.J., Liu S.L., Shang C.J. (2016). Micromechanical Behavior and Failure Mechanism of F/B Multi-phase High Performance Steel. J. Iron Steel Res. Int..

[5] Varshney A., Sangal S., Kundu S., Mondal K. (2016). Super strong and highly ductile low alloy multi-phase steels consisting of bainite, ferrite and retained austenite. Mater. Des..

[6] Saeidi N., Ekrami A. (2009). Comparison of mechanical properties of martensite/ferrite and bainite/ferrite dual phase 4340 steels. Mater. Sci. Eng. A.

[7] Bakhtiari R., Ekrami A. (2009). The effect of bainite morphology on the mechanical properties of a high bainite dual phase (HBDP) steel. Mater. Sci. Eng. A.

[8] Guo H., Gao G., Gui X., Misra R.D.K., Bai B. (2016). Structure-property relation in a quenched-partitioned low alloy steel involving bainite transformation. Mater. Sci. Eng. A.

[9] Gui X., Gao G., Guo H., Zhao F., Tan Z., Bai B. (2017). Effect of bainitic transformation during BQ&P process on the mechanical properties in an ultrahigh strength Mn-Si-Cr-C steel. Mater. Sci. Eng. A.

[10] Min J., Lin J., Min Y., Li F. (2012). On the ferrite and bainite transformation in isothermally deformed 22MnB5 steels. Mater. Sci. Eng. A.

[11] Abdollah-Zadeh A., Salemi A., Assadi H. (2008). Mechanical behavior of CrMo steel with tempered martensite and ferrite-bainite-martensite microstructure. Mater. Sci. Eng. A.

[12] Murari F.D., Da Costa E., Silva A.L.V., De Avillez R.R. (2015). Cold-rolled multi-phase boron steels: Microstructure and mechanical properties. J. Mater. Res. Technol..

[13] Hug E., Martinez M., Chottin J. (2015). Temperature and stress state influence on void evolution in a high-strength dual-phase steel. Mater. Sci. Eng. A.

[14] Han S.H., Choi S.H., Choi J.K., Seong H.G., Kim I.B. (2010). Effect of hot-rolling processing on texture and r-value of annealed dual-phase steels. Mater. Sci. Eng. A.

[15] Akbarpour M.R., Ekrami A. (2008). Effect of ferrite volume fraction on work hardening behavior of high bainite dual phase (DP) steels. Mater. Sci. Eng. A.

[16] Golling S., Östlund R., Oldenburg M. (2016). A study on homogenization methods for steels with varying content of ferrite, bainite and martensite. J. Mater. Process. Technol..

[17] Zhao Z.P., Qiao G.Y., Tang L., Zhu H.W., Liao B., Xiao F.R. (2016). Fatigue properties of X80 pipeline steels with ferrite/bainite dual-phase microstructure. Mater. Sci. Eng. A.

[18] Ormsuptave N., Uthaisangsuk V. (2017). Modeling of bake-hardening effect for fine grain bainite-aided dual phase steel. Mater. Des..

[19] Ishikawa N., Yasuda K., Sueyoshi H., Endo S., Ikeda H., Morikawa T., Higashida K. (2015). Acta Materialia Microscopic deformation and strain hardening analysis of ferrite–Bainite dual-phase steels using micro-grid method. Acta Mater..

[20] Saeidi N., Ekrami A. (2010). Impact properties of tempered bainite–ferrite dual phase steels. Mater. Sci. Eng. A.

[21] Wang K., Tan Z., Gao G., Gao B., Gui X., Misra R.D.K., Bai B. (2016). Microstructure-property relationship in bainitic steel: The effect of austempering. Mater. Sci. Eng. A.

[22] Magnabosco R., Matheisen A.S., Lima C.G. (1999). Anisotropy Indexes Determination in HSLA Steels.

[23] Ashrafi H., Shamanian M., Emadi R., Saeidi N. (2017). A novel and simple technique for development of dual phase steels with excellent ductility. Mater. Sci. Eng. A.

[24] Toribio J., González B., Matos J.C. (2013). Strength anisotropy and mixed mode fracture in heavily drawn pearlitic steel. Fatigue Fract. Eng. Mater. Struct..

[25] Komissarova L.A., Lipyanko I.A., Prusakov B.A., Surin A.I., Kruglov B.A. (1984). Anisotropy in mechanical properties of constructional steel. Met. Sci. Heat Treat..

[26] Nasser A., Yadav A., Pathak P., Altan T. (2010). Determination of the flow stress of five AHSS sheet materials (DP 600, DP 780, DP 780-CR, DP 780-HY and TRIP 780) using the uniaxial tensile and the biaxial Viscous Pressure Bulge (VPB) tests. J. Mater. Process. Technol..

[27] Goto S., Kami C., Kawamura S. (2015). Effect of alloying elements and hot-rolling conditions on microstructure of bainitic-ferrite/martensite dual phase steel with high toughness. Mater. Sci. Eng. A.

[28] Wang H.S., Kuo Y.L., Kuo C.M., Wei C.N. (2016). Microstructural evolution and mechanical properties of hot isostatic pressure bonded CM 247LC superalloy cast. Mater. Des..

[29] Xie B., Cai Q., Yu W., Xu S., Wang B. (2016). A Flow Stress Model for High Strength Steels with Low Carbon Bainite Structure. J. Iron Steel Res. Int..

[30] Cao J., Li F.G., Ma X.K., Sun Z.K. (2017). Tensile stress–strain behavior of metallic alloys. Trans. Nonferrous Met. Soc. China Engl. Ed..

[31] Colla V., De Sanctis M., Dimatteo A., Lovicu G., Solina A., Valentini R. (2009). Strain Hardening Behavior of Dual-Phase Steels. Metall. Mater. Trans. A.

[32] Zhang W., Wu J., Wen Y., Ye J., Li N. (2010). Characterization of different work hardening behavior in AISI 321 stainless steel and Hadfield steel. J. Mater. Sci..

[33] Bergström Y., Granbom Y., Sterkenburg D. (2010). A Dislocation-Based Theory for the Deformation Hardening Behavior of DP Steels: Impact of Martensite Content and Ferrite Grain Size. J. Metall..

[34] Lilehkoohi A.H., Faieza A.A., Sahari B.B., Nuraini A.A., Halali M. (2013). Effect of material on crashworthiness for side doors and B pillar subjected to euro NCAP side impact crash test. Adv. Sci. Lett..

[35] Soares G.C., Gonzalez B.M., Santos L.D.A. (2017). Strain hardening behavior and microstructural evolution during plastic deformation of dual phase, non-grain oriented electrical and AISI 304 steels. Mater. Sci. Eng. A.

[36] Maciejewski K., Ghonem H. (2014). Isotropic and kinematic hardening as functions of gamma prime precipitates in a nickel-based superalloy. Int. J. Fatigue.

[37] Tian C., Ponge D., Christiansen L., Kirchlechner C. (2020). On the mechanical heterogeneity in dual phase steel grades: Activation of slip systems and deformation of martensite in DP800. Acta Mater..

[38] Gao H., Huang Y. (2003). Geometrically necessary dislocation and size dependent plasticity. Scr. Mater..

[39] Rezvanian O., Zikry M.A., Rajendran A.M. (2007). Statistically stored, geometrically necessary and grain boundary dislocation densities: Microstructural representation and modelling. Proc. R. Soc. A Math. Phys. Eng. Sci..

[40] Arsenlis A., Parks D.M. (1999). Crystallographic aspects of geometrically-necessary and statistically-stored dislocation density. Acta Mater..

[41] Wu H.Y., Yan J.C., Tsai H.H., Chiu C.H., Zhou G.Z., Lin C.F. (2010). Tensile flow and strain-hardening behaviors of dual-phase Mg-Li-Zn alloy thin sheets. Mater. Sci. Eng. A.

[42] Tsuchida N., Tomota Y., Moriya H., Umezawa O., Nagai K. (2001). Application of the Kocks-Mecking model to tensile deformation of an austenitic 25Cr-19Ni steel. Acta Mater..

[43] Xie B.S., Cai Q.W., Yu W., Cao J.M., Yang Y.F. (2014). Effect of tempering temperature on resistance to deformation behavior for low carbon bainitic YP960 steels. Mater. Sci. Eng. A.

